# Expression of Insulin-Like Growth Factor Binding Protein-5 (*IGFBP5*) Reverses Cisplatin-Resistance in Esophageal Carcinoma

**DOI:** 10.3390/cells7100143

**Published:** 2018-09-20

**Authors:** Dessy Chan, Yuanyuan Zhou, Chung Hin Chui, Kim Hung Lam, Simon Law, Albert Sun-chi Chan, Xingshu Li, Alfred King-yin Lam, Johnny Cheuk On Tang

**Affiliations:** 1 State Key Laboratory of Chemical Biology and Drug Discovery, Lo Ka Chung Centre for Natural Anti-cancer Drug Development, Department of Applied Biology and Chemical Technology, The Hong Kong Polytechnic University, Hong Kong, China; dessychan@gmail.com (D.C.); yuanyuan.09.zhou@connect.polyu.hk (Y.Z.); chui.ch@gmail.com (C.H.C.), kim.hung.lam@polyu.edu.hk (K.H.L.); 2 Department of Surgery, Li Ka Shing Faculty of Medicine, The University of Hong Kong, Hong Kong, China; slaw@hku.hk; 3 School of Pharmaceutical Sciences, Sun Yat-sen University, Guangzhou 510006, China; chenxz3@mail.sysu.edu.cn; 4 Griffith Medical School, Griffith University, Gold Coast, QLD 4222, Australia

**Keywords:** cisplatin resistance, esophageal squamous cell carcinoma, insulin-like growth factor binding protein-5

## Abstract

Cisplatin (CDDP) is one of the front-line chemotherapeutic drugs used in the treatment of esophageal squamous cell carcinoma (ESCC). Occurrence of resistance to CDDP has become one of the main challenges in cancer therapy. In this study, the gene expression profile of CDDP-resistant ESCC cells was investigated and molecular approaches were explored in an attempt to reverse the CDDP resistance. A CDDP-resistant SLMT-1/CDDP1R cell line was established from SLMT-1 cells by subculturing in the medium containing an increasing concentration of CDDP (0.1–1μg/mL). Mitochondrial (MTS) cytotoxicity assay, cell proliferation assay and cell morphology were used to assess the acquisition of cisplatin-resistance. The most differentially expressed gene in SLMT-1/CDDP1R cells was identified by cDNA microarray analysis compared with the parental SLMT-1 cells and validated by quantitative real-time polymerase chain reaction (qPCR). Association between expression of the most differentially expressed target gene to cisplatin-resistance was verified by RNA interference. An attempt to reversecisplatin-resistance phenotypes was made by using the vector expressing the most downregulated target gene in the CDDP-resistant cells. A CDDP-resistant ESCC cell line, SLMT-1/CDDP1R, was established with 2.8-fold increase CDDP-resistance (MTS_50_ = 25.8 μg/mL) compared with the parental SLMT-1 cells. cDNA microarray analysis revealed that *IGFBP5* showed the highest level of downregulation in SLMT-1/CDDP1R cells compared with the parental SLMT-1 cells. Suppression of *IGFBP5* mediated by *IGFBP5*-targeting siRNA in parental SLMT-1 cells confirmed that *IGFBP5* suppression in ESCC cells would induce CDDP-resistance. More importantly, upregulation of *IGFBP5* using *IGFBP5* expression vector reduced cisplatin-resistance in SLMT-1/CDDP1R cells by 41%. Thus, our results demonstrated that *IGFBP5* suppression is one of the mechanisms for the acquisition of cisplatin-resistance in ESCC cells. Cisplatin-resistance phenotype can be reversed by increasing the expression level of *IGFBP5*. The overall findings of this study thus offered a new direction for reversing the CDDP resistance in ESCC and possibly in other cancer types with further investigations in future.

## 1. Introduction

Esophageal squamous cell carcinoma (ESCC) is an aggressive cancer with high mortality rate [1]. Although many therapeutic strategies have been adopted, the overall 5-year survival rateremained low, at around 20% [2]. Cisplatin (CDDP) is used as one of the key chemotherapeutic drugs in the front-line treatment of ESCC. CDDP-based therapeutic approaches always gain initial therapeutic success. However, tumors eventually develop chemoresistance to CDDP, which becomes one of the major problems with CDDP-based treatment [3]. Thus, elucidation of mechanisms leading to the development of cisplatin-resistance in ESCC would hopefully offer possible solutions to reverse CDDP-resistance and improve efficacy of CDDP-treatment.

In vitro studies on solid cancer cell lines revealed that mechanisms of cisplatin-resistance are multi-factorial [4]. Prominent mechanisms involve various biological regulatory processes, such as decreased drug transport, increased cellular detoxification by cellular thiols glutathione (GSH) and metallothionein (MT), changes in DNA repair involving increased nucleotide excision repair and/or loss of mismatch repair, increased tolerance of DNA adducts, and defeated apoptotic cell death pathway [5]. Recent findings revealed that hyperactivation of insulin-like growth factor (IGF) signaling was associated with reduced sensitivity to cisplatin-based chemotherapy in several types of cancers including ESCC [6],ovarian cancer [7], lung cancer [8] and mesothelioma [9]. Acting as the inhibitory regulation-proteins in IGF signaling pathway [10], insulin-like growth factor binding proteins (IGFBPs) have been shown to be associated with the sensitivity to CDDP. Inhibition of IGF signaling by IGFBP3 was shown to result in increased sensitivity to CDDP in CDDP-resistant lung cancer cells [8]. In spite of the fact that many studies have characterized the roles of six IGFBPs (IGFBP1-6) in the IGF signaling pathway [10,11,12], studies on the acquisition of CDDP-resistance mediated by IGFBPs are scanty. Moreover, other candidate proteins have also been reported to be involved in tumor growth and/or therapeutic responses; examples include, but not limited to, insulin-like growth factor binding protein 5 (IGFBP5) [13], major histocompatibility complex, class II, DQ alpha 2 (HLA-DQA2), [14] carboxypeptidase E (PE) [15], and PLXDC2 (CDNAFLJ45742fis) [16]. In the present study, a CDDP-resistance ESCC cell line was established and the changes in gene expression profile were identified using cDNA microarray with validation in attempt to determine the most differentially expressed gene with CDDP-resistance. To derive a novel approach for molecular therapy against CDDP-resistance, the most differentially downregulated gene was further examined with RNA interference in the parental ESCC cells to determine its association with the acquired CDDP-resistance and evaluate for the feasibility to be the target gene for reversing CDDP-resistance in ESCC. The cell line models involved the use of an ESCC cell line SLMT-1 established by our research team [17] and the derived SLMT-1/CDDP1R with established CDDP resistance in the present study. The overall results of the present study will provide a new direction for reversing the CDDP-resistance in ESCC and possibly in other cancers with future investigations.

## 2. Results

### 2.1. Evaluation forCisplatin-Resistance

**Figure 1:**
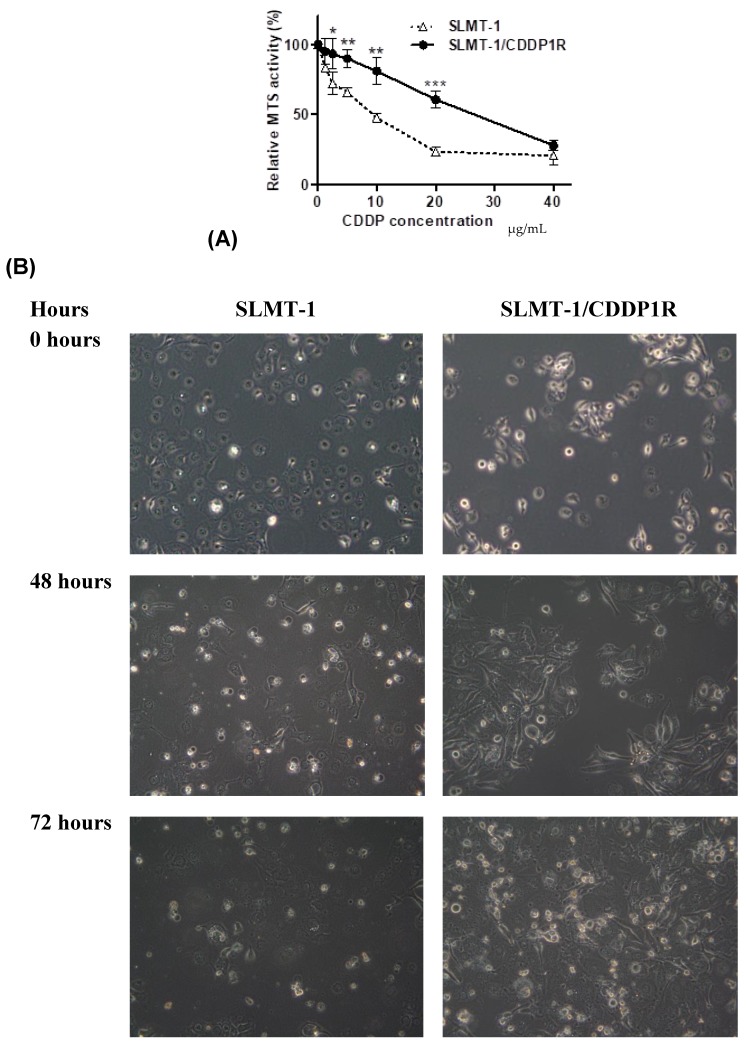

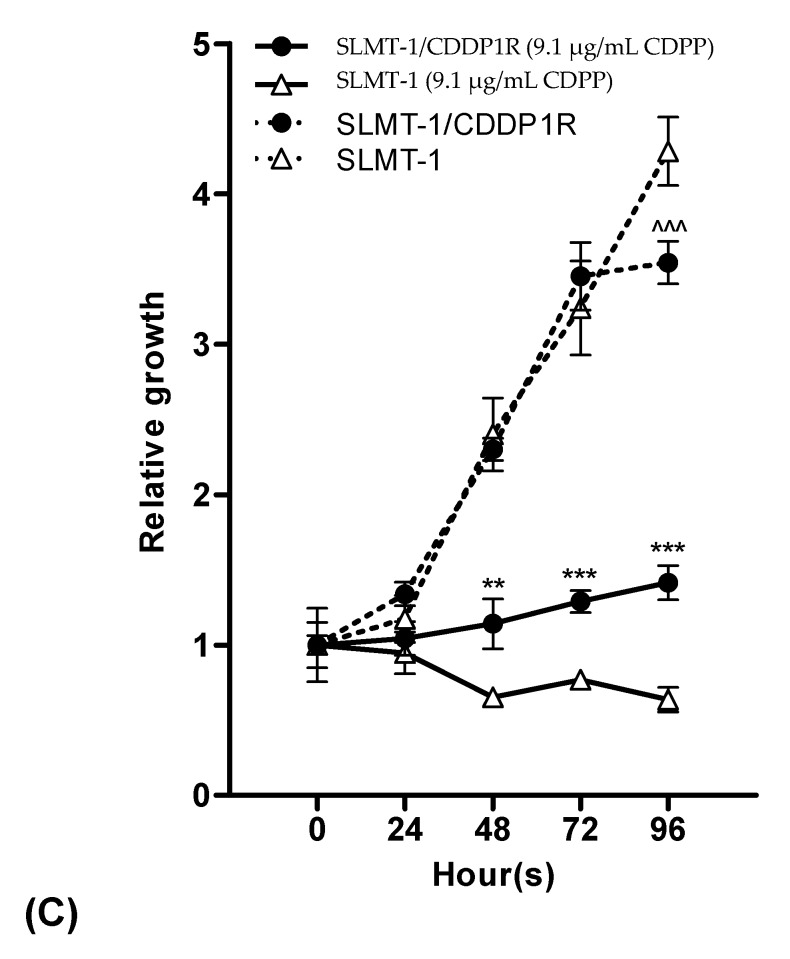
Evaluation for cisplatin-resistance. (**A**) Relative mitochondrial (MTS) activities of SLMT-1 and SLMT-1/CDDP1R cells after 48 h treatment with cisplatin (CDDP) at different concentrations (0, 1.25, 2.5, 5, 10, 20 and 40 μg/mL). Relative MTS activities were expressed as means ± standard error compared with the MTS activities at 0 hand analyzed using one-way ANOVA. The MTS activities of SLMT-1/CDDP1R was compared to SLMT-1 with * *p* < 0.05, ** *p* < 0.01 and *** *p* < 0.001. (**B**) Morphology of SLMT-1 and SLMT-1/CDDP1R cells after culturing in medium with 9.1 μg/mL CDDP for 0, 48 and 72 h. The SLMT-1 cells with less CDDP-resistance showed more roundness, shrinkage and detachment, indicating the cytotoxic effects of CDDP. (**C**) Relative growth of SLMT-1 and SLMT-1/CDDP1R cultured in medium with or without 9.1 μg/mL CDDP. Relative growth was expressed as means ± standard error compared with the respective MTS activities at 0 hand analyzed using one-way ANOVA. SLMT-1/CDDP1R (9.1 μg/mL CDDP) was compared to SLMT-1 (9.1 μg/mL CDDP) with ** *p* < 0.01 and *** *p* < 0.001.SLMT-1/CDDP1R was compared to SLMT-1 with ^^^ *p* < 0.001. SLMT-1/CDDP1R grew in a significantly higher rate than SLMT-1 in medium with 9.1 μg/mL CDDP.

The sensitivity of SLMT-1 and SLMT-1/CDDP1R cells to CDDP was examined by mitochondrial (MTS) viability assay and cell morphology. As shown in Figure 1A, SLMT-1/CDDP1R showed remarkably higher relative MTS activity than SLMT-1 after 48 h treatment with cisplatin at concentration of 2.5–20 μg/mL MTS proliferation assay indicates the changes in the mitochondrial activity (MTS activity) and hence the cell viability can be evaluated [18]. MTS_50_ was defined as the concentration of the test agent required to reduce the MTS activity by 50% when compared with the untreated control. The MTS_50_ in SLMT-1 and SLMT-1/CDDP1R cells were 9.1 μg/mL and 25.8 μg/mL respectively. SLMT-1/CDDP1R cells showed a 2.8-fold increase in resistance to CDDP compared with parental SLMT-1 cells at 48 h. When SLMT-1 and SLMT-1/CDDP1R cells were cultured in the medium containing 9.1 μg/mL CDDP (MTS_50_ value of parental SLMT-1 to CDDP), SLMT-1 cells shrank, became rounded-up and showed increased detachment after 48 h but SLMT-1/CDDP1R cells showed increase in confluence from 0to 72 h (Figure 1B), indicating the cytotoxic effects of the test agent (CDDP) were induced in the cancer cells as described [19]. Figure 1C shows that proliferation of parental SLMT-1 cells was suppressed under 9.1 μg/mL CDDP by 9 times at 96 h of incubation, while the proliferation of SLMT-1/CDDP1R cells was suppressed only by 2.3 times under 9.1 μg/mL CDDP at 96 h of incubation. The results indicated that the SLMT-1/CDDP1R cells showed more CDDP-resistance than the parental SLMT-1 cells. The MTS_50_ values for CDDP from the panel of ESCC and non-tumor cells are summarized in Table 1.

### 2.2. Differentially Expressed Genes in SLMT-1 and SLMT-1/CDDP1R

**Figure 2:**
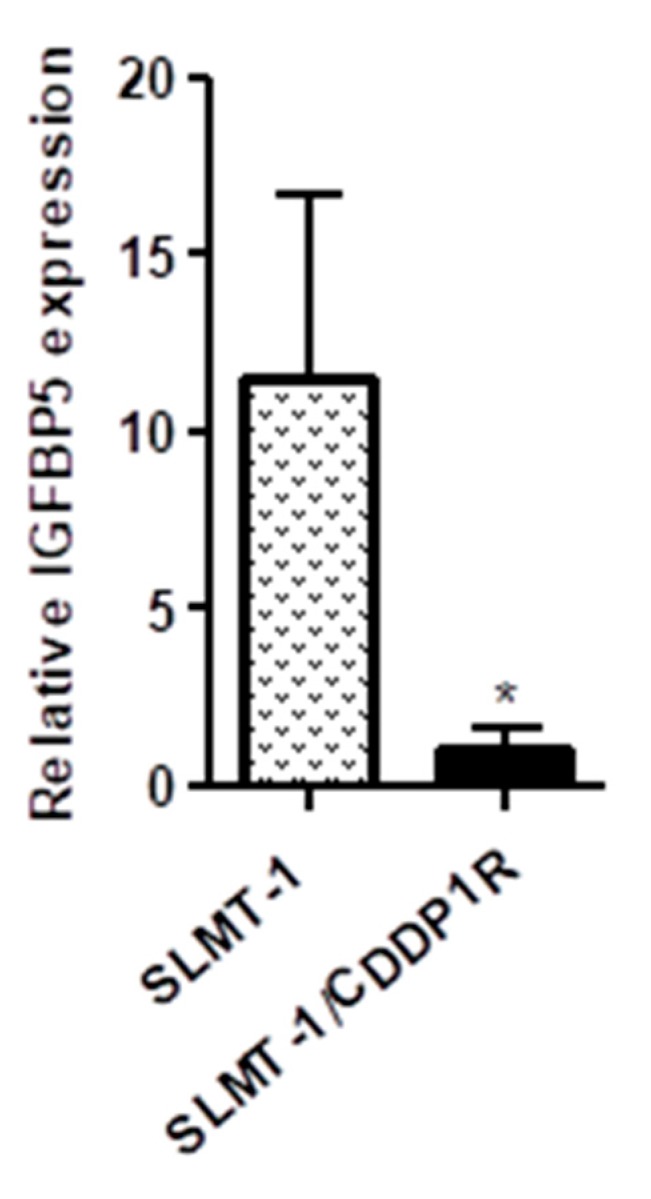
Validation of insulin-like growth factor binding protein 5 (*IGFBP5*) expression by quantitative real-time polymerase chain reaction (qPCR) analysis. Relative expression level of *IGFBP5* in SLMT-1/CDDP1R was significantly lower than SLMT-1.Relative *IGFBP5* expression in SLMT-1/CDDP1R was determined by comparing with SLMT-1, after normalized with the expression of β-actin. * *p* < 0.05.

Compared with the parental SLMT-1 cells, *IGFBP5*, *HLA-DQA2*, *PE* and *CDNA FLJ45742fis* were found to be the most downregulated candidates (from −14.09 to −43.48) in SLMT-1/CDDP1R using the microarray analysis (Table 1). *LINC00520*, *SLITRK6*, *LOC100506377* and *COL15A1* were the most upregulated genes(from 8.79 to 16.89 folds) in SLMT-1/CDDP1R. *IGFBP5* showed the highest fold-change (43.48 folds) of downregulation and thus was selected as the target gene for the subsequent study for reversing the CDDP-resistance. Quantitative real-time polymerase chain reaction (qPCR) analysis was performed to validate the downregulation of *IGFBP5* in SLMT-1/CDDP1R. As shown in Figure 2, the relative expression level of *IGFBP5* in SLMT-1/CDDP1R cells was significantly lower than that of SLMT-1 parental cells and it was in line with the data of microarray analysis that *IGFBP5* showed its downregulation in SLMT-1/CDDP1R cells.

### 2.3. IGFBP-5 Downregulation AcquiresCisplatin-Resistance

**Figure 3:**
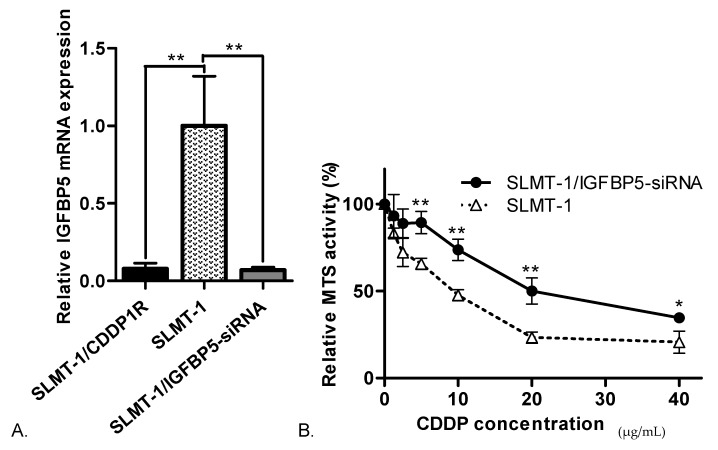
Association of IGFBP-5 downregulation and cisplatin-resistance. (**A**) Relative expression levels of *IGFBP5* in SLMT-1/IGFBP5-siRNA cells was significantly lower than SLMT-1 cells. Relative *IGFBP5* expression levels were determined by comparing with SLMT-1 cells, after normalized with expression of β-actin. ** *p* < 0.01. (**B**) Relative MTS activities of SLMT-1 and SLMT-1/IGFBP5-siRNAcells after 48 h treatment with cisplatin at different concentrations (0, 1.25, 2.5, 5, 10, 20 and 40 μg/mL). Relative MTS activities were expressed as means ± standard error compared with the MTS activities at zero CDDP concentration and analyzed using one-way ANOVA. SLMT-1/IGFBP5-siRNA was compared to SLMT-1 with * *p* < 0.05 and ** *p* < 0.01 IGFBP5-siRNA was effective in inducing the significant proliferation of SLMT-1 cells under the CDDP treatment.

To further study the role of *IGFBP5* in acquiring cisplatin-resistance, siRNA targeting *IGFBP5* was transfected into SLMT-1 parental cells. As shown in Figure 3A, expression level of *IGFBP5* was significantly reduced by 10 times using siRNA-based RNA interference. Sensitivity of SLMT-1/IGFBP5-siRNA cells to CDDP was examined by MTS cytotoxicity assay (Figure 3B). SLMT-1/IGFBP5-siRNA cells showed significantly higher relative MTS activity than SLMT-1 after 48 h cisplatin treatment at concentration of 5–40 μg/mL. MTS_50_ of SLMT-1/IGFBP5-siRNA is 20.5 μg/mL, which is over 2.3-fold increase in resistance to cisplatin compared with parental SLMT-1. And the increase in cisplatin-resistance in SLMT-1/IGFBP5-siRNA was comparable to that of SLMT-1/CDDP1R (with MTS_50_ = 25.8 μg/mL).

### 2.4. Upregulation of IGFBP5 Reverses Cisplatin-Resistance

**Figure 4:**
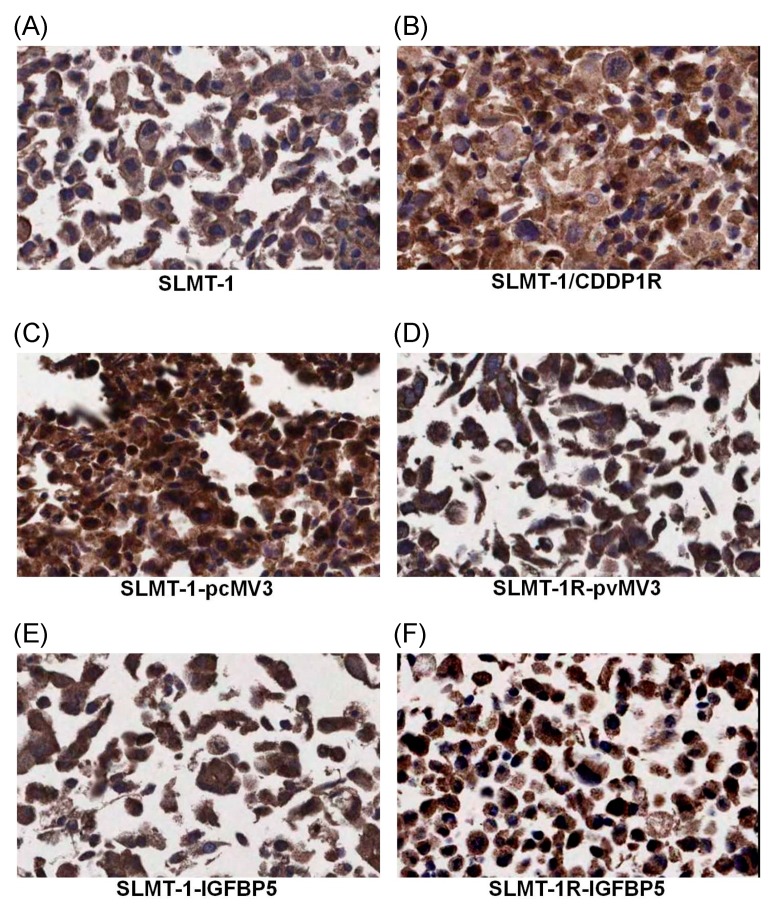
Immunohistochemical staining of Myc-tagged pcMV3 vector in (**A**) SLMT-1. (**B**) SLMT-1/CDDP1R. (**C**) SLMT-1-pcMV3. (**D**) SLMT-1R-pcMV3. (**E**) SLMT-1-IGFBP5 and (**F**) SLMT-1R-IGFBP5 cells showed the transfection efficiency of IGFBP5 and pcMV3 mock vector. More positive staining nuclear and cytoplasmic signals (>50%) were observed in SLMT-1-pcMV3, SLMT-1R-pcMV3, SLMT-1-IGFBP5 and SLMT-1R-IGFBP5 than the untransfected SLMT-1 and SLMT-1/CDDP1R cells. Original magnification: ×400.

**Figure 5:**
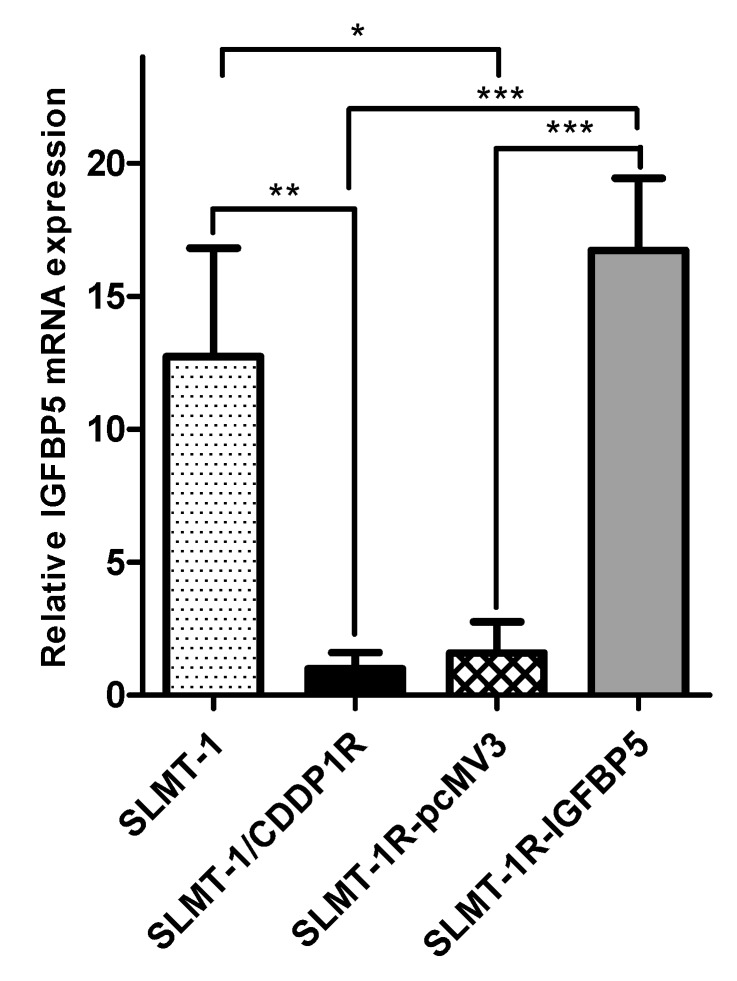
Relative mRNA expression levels of *IGFBP5* in SLMT-1 parental cells, SLMT-1/CDDP1R, SLMT-1R-pcMV3 and SLMT-1R-IGFBP5.Significantly higher expression level of *IGFBP5* was observed in SLMT-1R-IGFBP5 compared with the SLMT-1/CDDP1R and SLMT-1R-pcMV3 cells after normalization with the expression of β-actin in qPCR. *** *p <* 0.001, ** *p <* 0.05, * *p <* 0.01.

**Figure 6:**
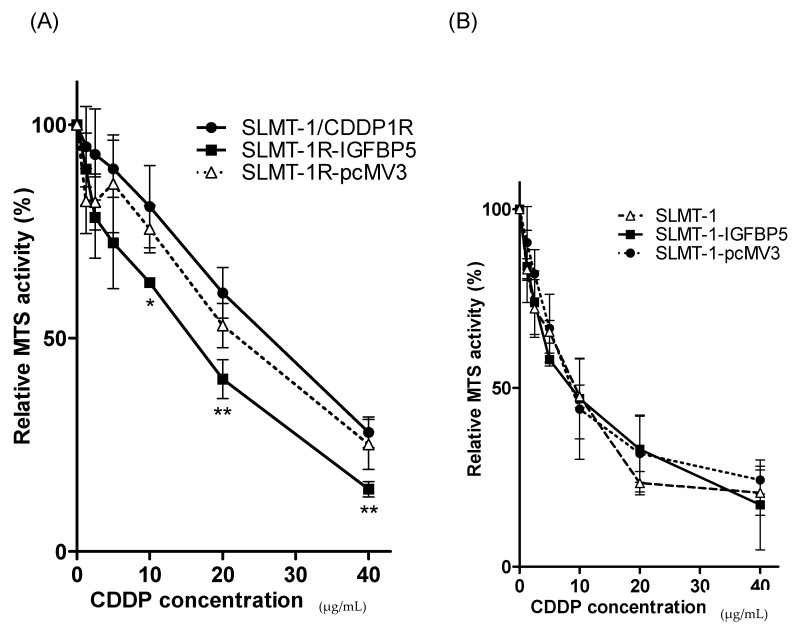

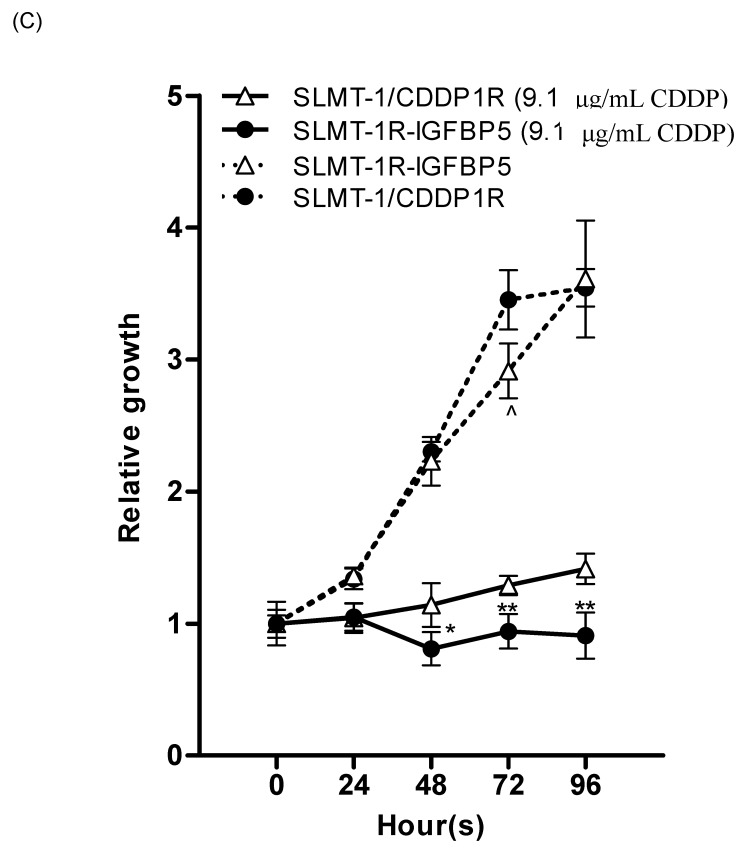
*IGFBP5* expression vector reversed cisplatin-resistance. (**A**) Relative MTS activity of SLMT-1/CDDP1R, SLMT-1R-IGFBP5 and SLMT-1R-pcMV3 cells. Relative MTS activities were expressed as means ± standard error compared with the MTS activities at zero CDDP concentration and analyzed using one-way ANOVA. SLMT-1R-IGFBP5 was compared to SLMT-1R-pcMV3 with* *p* < 0.05 and ** *p* < 0.01. SLMT-1R-IGFBP5 was more sensitive to CDDP than SLMT-1R-pcMV3 and SLMT-1/CDDP1R. (**B**) Relative MTS activity of SLMT-1, SLMT-1-IGFBP5 and SLMT-1-pcMV3 cells after 48 h treatment with cisplatin at different concentrations compared to SLMT-1. SLMT-1, SLMT-1-IGFBP5 and SLMT-1-pcMV3 cells showed similar sensitivity to cisplatin. (**C**) Relative growth of SLMT-1/CDDP1R and SLMT-1R-IGFBP5 cultured in medium with or without 9.1 μg/mL cisplatin. Relative growth was expressed as means ± standard error compared with the MTS activities at zero hour and analyzed using one-way ANOVA. SLMT-1R-IGFBP5 (9.1 μg/mL CDDP) was compared to SLMT-1/CDDP1R (9.1 μg/mL CDDP) with * *p* < 0.01 and ** *p* < 0.001. SLMT-1R-IGFBP5 was compared to SLMT-1/CDDP1R with ^ *p* < 0.001. Growth of SLMT-1R-IGFBP5 cells was significant inhibited than SLMT-CDDP/1R in the medium with 9.1μg/mL CDDP, demonstrating the reversal of CDDP-resistance by *IGFBP5* expression vector.

It has been reported that the wild type protein of IGFBP5 can be localized in cytoplasm and nucleus [20,21]. The results of IHC staining (Figure 4C–F) showed the effective transfection of Myc-tagged IGFBP5/pcMV3-C-Myc vector and Myc-tagged pcMv/hygro-negative control vector in all the four transfected cell lines (SLMT-1-pcMV3 (Figure 4C), SLMT-1R-pcMV3 (Figure 4D), SLMT-1-IGFBP5 (Figure 4E) and SLMT-1R-IGFBP5 (Figure 4F)). More than 50% of cells in these cell lines showed more positive staining signals (dark brown) in cytoplasm and nuclei. The SLMT-1 and SLMT-1/CDDP1R cells (Figure 4A,B), which were not transfected with Myc-tagged vector, showed lesser positive staining signals. Moreover, as shown in Figure 5, the transfection of *IGFBP5* expression vector into SLMT-1/CDDP1R cells remarkably increased the expression of *IGFBP-5* in SLMT-1R-IGFBP5 cells (*p* = 0.0006). Transfection of control vector (pcMV3) into SLMT-1/CDDP1R cells (to give SLMT-1R-pcMV3) did not influence the expression of *IGFBP5* (*p* = 0.4790). As shown in Figure 6A, MTS_50_ of SLMT-1R-IGFBP5 was 15.3 μg/mL, which was significantly lower than that of SLMT-1/CDDP1R (25.8 μg/mL). Upregulation of *IGFBP5* by IGFBP5/pcMV3-C-Myc vector in SLMT-1/CDDP1R was able to reduce cisplatin-resistance by 41%. MTS_50_ of SLMT-1R-pcMV3 was 21.8 μg/mL which had no significant difference (*p* = 0.4790) with that of SLMT-1/CDDP1R(25.8 μg/mL). MTS_50_ of SLMT-1-pcMV3 and SLMT-1-IGFBP5 were 9.7μg/mL and 9.6μg/mL respectively (Figure 6B), which showed no significant differences (*p* = 0.4590 and *p* = 0.7411) with MTS_50_ of SLMT-1 (9.1 μg/mL). These indicated that transfection of *IGFBP5* expression vector and mock vector in parental SLMT-1 did not affect sensitivity of cisplatin-sensitive SLMT-1 to cisplatin. Reversal of cisplatin resistance in SLMT-1R-IGFBP5 was demonstrated in cell proliferation assay. As shown in Figure 6C, proliferation of SLMT-1R-IGFBP5 cells was suppressed when it was cultured in medium containing 9.1 μg/mL CDDP (MTS_50_ value of parental SMLT-1 to CDDP). Compared to the proliferation of SLMT-1/CDDP1R cells, significant differences were found at the time points of 48, 72 and 96 h, implying that SLMT-1R-IGFBP5 was more CDDP-sensitive than SLMT-1/CDDP1R at these time points. Nevertheless, SLMT-1R-IGFBP5 cells were able to proliferatein similar rate as SLMT-1/CDDP1R in culture condition without cisplatin. Proliferation assay further demonstrated that SLMT-1R-IGFBP5 cells showed a significant reduction in cisplatin resistance (Figure 6A).

## 3. Discussion

The anti-cancer drug cisplatin belongs to the category of alkylator which causes DNA alkylation to induce cytotoxicity in cancer cells [4,5] and is an effective front-line chemotherapeutic drug against cancer that causes DNA damagefollowed by apoptosis [22]. The mechanisms of acquiring cisplatin resistance are multifactorial and may be unique to different types of cancers [5,23]. Occurrence of resistance to cisplatin isthe mainchallenge in the chemotherapy for ESCC and other cancers. Several mechanisms contributing to resistance to cisplatin have been suggested and they include blocking the transduction of DNA damaging signal and/or apoptotic signal, development of DNA repairing mechanisms, as well as efflux of cisplatin from cancer cells [5]. Thus more understanding about the mechanisms of CDDP resistance is definitely helpful to develop new strategies for cancer therapy at a molecular level.

In the present study, cisplatin-resistant ESCC cell line SLMT-1/CDDP1R was obtained by repeatedly incubating SLMT-1 cells with increasing doses of cisplatin. And the differential expression of genes in the cisplatin-resistant cell line relative to parental cells was identified using cDNA microarray technology. A similar approach was adopted in some previous studies for identifying the potential molecular targets in ESCC cells with cisplatin-resistance. A cisplatin-resistant subline for ESCC cell line YES-2 (Yamamoto Esophageal Squamous-2; Japanese origin) was established and characterized the cisplatin-resistant YES-2 cell line with decreased cisplatin accumulation and frequent under-expression of genes encoding ribosome-related proteins [24]. It was revealed that the increase in autophagy activity in cisplatin-resistant EC109 (Esophgeal Cancer; Chinese origin) cells compared with the parental EC109 and inhibition on autophagy was able to enhance the cytotoxic effect of cisplatin on the resistant cell line [25].

Moreover, in the present study, significant downregulation of *IGFBP5* was observed in SLMT-1 cells with cisplatin-resistance by microarray analysis and verified by qPCR. The causal link of downregulation of *IGFBP5* and acquisition of cisplatin-resistance was confirmed by using siRNA-based RNA interference. To the best of our knowledge, this is the first study which demonstrated the downregulation of *IGFBP5* leading to acquisition of cisplatin-resistance in ESCC. Another member of IGFBPs family, *IGFBP3*, was also shown to have similar effect in the study conducted andover-expressing *IGFBP3* enhanced sensitivity of KYSE30 (Kyoto, YShimada, Esophageal; Japanese origin) cells to cisplatin, and knocking-down of *IGFBP3* by specific siRNA reduced sensitivity of KYSE30 to cisplatin [26]. In order to assess whether downregulation of *IGFBP5* results in hyperactiviation of the IGF-signaling pathway and in turn confers cisplatin-resistance to ESCC, investigation of the status of IGF-signaling in SLMT-1/CDDP1R compared with parental SLMT-1 is suggested in future studies. Furthermore, suppression of *IGFBP5* by specific siRNA resulted in the decrease in sensitivity to cisplatin treatment and the observation indicated the potential of reversal of cisplatin-resistance by restoring *IGFBP5* level in SLMT-1/CDDP1R cells. *IGFBP5* expression vector was thus transfected into SLMT-1/CDDP1R cells to induce expression of *IGFBP5* and was evaluated for the efficacy of reversing the resistance. Significant reduction in cell viability was shown in cytotoxicity assay of CDDP in cisplatin-resistant ESCC cells after transfection of *IGFBP5* expressing vector with *IGFBP5* expression. A similar approach of restoring expression of TNF family receptors and caspases resulting in enhanced sensitivity of cancer cells to chemotherapy was also reported as a protocol for gene therapy [27]. Thus, our overall findings offer the first report to indicate the potential application of increasing the expression of *IGFBP5* in restoring the CDDP sensitivity in ESCC cells.

IGFBPs are known fortheir inhibitory effect on IGF-stimulated activities by sequestering IGF away from IGF-R [10,28]. Hyperactivation of the IGF signaling pathway has been reported tolead to cisplatin-resistance in ovarian cancer [7] and lung cancer [8]. Improved cisplatin treatment outcome was observed in inhibition of IGF signaling pathway. Blockade of IGF signaling at IGF-receptor(IGF-1R) was found enhancing cisplatin-induced apoptosis in ESCC cells [29]. The use of monoclonal antibodies against IGF-1R accompanying cisplatin improved inhibitory efficacy in small cell lung cancer in vivo and in vitro in nude mice bearing the tumors [30]. The mechanism underlying the enhancing effect of cisplatin by blockade of IGF signaling pathway was not yet fully understood. However, some previous findings about the molecular of action of the *IGF-1* signaling pathway provided some ideas for it. It was found that the DNA repair pathway, *p38 MAP* kinase signaling pathway, is mediated by *IGF-1* in fibroblast cells. As the anti-tumor effect of cisplatin relies on its DNA damage properties, DNA repairing mediated by *IGF-1* might lead to reduced sensitivity to cisplatin [31]. In addition, *IGF-1* rescued cells from apoptosis by inducing *p53* protein degradation upon DNA damage [32]. *p53* is a key regulator in DNA damaging signaling and degradation of p53 protein might aid the damaged cells to escape from apoptosis, and thus acquired cisplatin-resistance [33].

To conclude, our report described for the first time the down regulation of *IGFBP5* that conferred CDDP resistance in ESCC and the potential role of restoring the CDDP sensitivity in ESCC cells through increasing the expression of *IGFBP5*. This approach offers the potential prospect of overcoming cisplatin-resistance if the work can be extended to the other human cancer types as the approach of gene therapy in future. The possible long-term benefits include the improved efficacy of cisplatin and thus the reduction of the overall treatment cost for cancer patients.

## 4. Material and Methods

### 4.1. Establishment of an ESCC Cell Line with Resistance to Cisplatin

The ESCC cell line SLMT-1 [17] was maintained in minimum essential medium alpha (MEMα, Gibco, NY, USA) supplemented with 20% FBS, 100 μg/mL penicillin and 100 unit/mL streptomycin at 37 °C in a humidified incubator with 5% CO_2_. The cisplatin-resistant cell line, SLMT-1/CDDP1R, was established from the SLMT-1 cells by repeatedly subcultured under an increasing concentration of cisplatin (CDDP) (Sigma-Aldrich, St Louis, MO, USA)starting from 0.1 μg/mL to final concentration of 1.00 μg/mL.

### 4.2. Cell Proliferation Assay

The cytotoxic effect induced by CDDP in SLMT-1 and SLMT-1/CDDP1R cells, and the proliferation of these two cell lines in media with or without CDDP were determined by the MTS ([3-(4,5-Dimethylthiazol-2-yl)-5-(3-carboxymethoxyphenyl)-2-(4-sulfophenyl)-2*H*-tetrazolium]) cell proliferation assay using CellTiter-96-AQueous One Solution Cell Proliferation Assay reagent (Promega, Fitchburg, WI, USA). The cells were plated at 5 × 10^3^ per well in flat-bottom 96-well plate in 100 μL of MEMα supplemented with 20% FBS and allowed to attach overnight. The cells were treated with CDDP in DMSO (0.01%) at concentration of 40, 20, 10, 5, 2.5 and 1.25 μg/mL for 48h. MTS_50_ (concentration of CDDP that caused 50% inhibition on the MTS activity) was determined as previously described by our group [34]. One the other hand, the proliferation of SLMT-1 and SLMT-1/CDDP1Rcells proliferation in the culturing media with or without 9.1 μg/mLCDDP (the MTS_50_ value as determined from the parental SLMT-1 cells) was monitored every 24 h for 96 h as previously described [35]. The cells were plated at 1 × 10^3^ per well in the 96-well plate in 100 μL of the respective culture medium. The medium was replaced with the one containing 9.1 μg/mL CDDP 24 h after seeding of cells and was monitored for a total of 96 h. Each assay was performed in triplicate. The MTS cytotoxicity assays using CDDP were also performed on the panel of ESCC and non-tumor cell lines to determine the MTS_50_ values.

### 4.3. Morphology Study

Morphology of SLMT-1 and SLMT-1/CDDP1R cells in culturing medium with or without 9.1 μg/mLCDDP was observed under microscope at 0, 48 and 72 h. Photos of cells were captured using light microscope (CKX41) (Olympus, Tokyo, Japan) with digital camera (DP71) (Olympus) at 200× magnification. The morphological appearance of the SLMT-1 and SLMT-1/CDDP1R cells was compared at each time point.

### 4.4. cDNA Microarray Analysis

The differentially expressed genes of parental SLMT-1 and SLMT-1/CDDP1R cells were identified by cDNA microarray analysisusing Human Genome U133 Plus 2.0 arrays (Affymetrix, Santa Clara, CA, USA) as previously described by our group [36]. The microarray signals were analyzed using Agilent Genespring GX (Agilent, Santa Clara, CA, USA) and Affymetrix GeneChip Operating Software (Version 1.4; Affymetrix, Santa Clara, CA, USA). The signals of the differentially expressed genes in SLMT-1/CDDP1R were compared with the parental SLMT-1 and the fold changes of up- or downregulated genes were determined.

### 4.5. Reverse Transcription Polymerase Chain Reaction (RT-PCR) and Quantitative Real-Time PCR (qPCR)

qPCR analysis was preformed to examine the expression level of *IGFBP5* in different cell lines. The total RNA of cells and patient samples was extracted using RNeasy Mini Kit (Qiagen, Venlo, Netherlands) and cDNAs were synthesized from total RNA using the GoScript™ Reverse Transcription System (Promega, Fitchburg, WI,, USA) according to the manufacturers’ instructions. The expression level of *IGFBP5* in the SLMT-1/CDDP1R cells was determined by qPCR analysis using Go Taq^®^ qPCR Master Mix (Promega, Fitchburg, WI, USA) and Thermo Scientific PikoReal Real-Time PCR System (Thermo Fisher Scientific, Waltham, MA, USA) according to the manufacturer’s protocol. cDNA (~2 μg) produced by reverse transcription from the RNA, wasamplified by using specific *IGFBP5* and β-actin gene primer pairs. Primers for IGFBP5: 5′-AACGAAAAGAGCTACCGCGA-3′ (forward) and 5′-CCGACAAACTTGGACTGGGT-3′ (reverse). Primers for β-actin: 5′-ACCTTCTACAATGAGCTGCG-3′ (forward) and 5′-CCTGGATAGCACGTACATGG-3′ (reverse). Relative *IGFBP5* expression in SLMT-1/CDDP1R was determined by comparing with parental SLMT-1 cells after being normalized with the expression of β-actin.

### 4.6. Suppression of IGFBP5 Expression by RNA Interference

To examine the effect of suppressing the expression of *IGFBP5* in SLMT-1 cells, siRNA targeting *IGFBP5* (Ambion, Waltham, MA, USA) was transfected into SLMT-1 cells. The siRNA sequences were 5′-GCAAGUCAAGAUCGAGAGATT-3′ (Sense) and 5′-UCUCUCGAUCUUGACUUGCTC-3′ (Antisense). SLMT-1 cells were seeded into a flat-bottom 96-well plate (SPL Life Sciences, Seoul, Korea) at a density of 2 × 10^3^ cells with 100μL of its culture medium. Transfection of the siRNA into the cells was conducted using Lipofectamine^®^ RNAiMAX Transfection Reagent (Invitrogen, Waltham, MA, USA) according to the manufacture’s instructions. The transfected SLMT-1/IGFBP5-siRNA cells together with the parental SLMT-1 and SLMT-1/CDDP1R cells were examined using qPCR analysis for the *IGFBP-5* expression, and MTS viability assay for the cytotoxicity effect of CDDPwas conducted with the procedures described above after incubating with the transfection medium for 48 h.

### 4.7. Restoring Expression Level of IGFBP5

*IGFBP5* expressing vector, *IGFBP5*/pcMV3-C-Myc (Sino Biological Inc., Beijing, China) was transfected into SLMT-1/CDDP1R and parental SLMT-1 cells to examine the effect of restoring expression level of *IGFBP5* on sensitivity to cisplatin. Myc tagged pcMV/hydro-negative control vector (Sino Biological Inc., Beijing, China) was transfected into SLMT-1/CDDP1R and parental SLMT-1 cells as controls. The transfection was conducted using FuGene^®^ HD transfection reagent (Promega, Fitchburg, WI, USA) according to the manufacture’s instructions. The transfected cells were selected using culture medium containing 50–400 μg/mL hygromycinB (Invitrogen, Waltham, MA, USA) and expanded to stable cell lines SLMT-1-R-IGFBP5 (SLMT-1/CDDP1R cells transfected with *IGFBP5* expressing vector), SLMT-1-IGFBP5 (SLMT-1 cells transfected with *IGFBP5* expressing vector), SLMT-1-pcMV3 (SLMT-1 cells transfected with control vector) and SLMT-1-R-pcMV3 (SLMT-1/CDDP1R cells transfected with control vector). The *IGFBP5* expression in cells was examined by qPCR analysis as described in the previous section. MTS proliferation assay was performed to evaluate the sensitivity of the transfected cells to CDDP.

### 4.8. Evaluation of the Transfection Efficiency by Immunohistochemical Staining

The paraffin-embedded cell-line blocks of SLMT-1/pcMV3, SLMT-1/IGFBP5, SLMT-1 R/pcMV3, SLMT-1 R/IGFBP5, SLMT-1 and SLMT-1/CDDP1Rwere prepared from cell pellets with approximately 5 × 10^5^ cells which had been formalin-fixed and wax-embedded as described [36]. Immunohistochemical staining was performed using DakoEnVision+system-HRP (Dako Corporation, Glostrup, Denmark) and myc-tag mouse monoclonal antibody (1 mg/mL, Sino Biological Inc., Beijing, China) was applied at a dilution factor of 1:8000 for overnight incubation at 4 °C as previously described [36]. The photos of staining samples were taken under microscope with the magnification of ×400.

### 4.9. Statistical Analysis

To describe the relative changes of gene expression level of the target gene compared with the reference gene, the comparative ΔΔCt method was applied for relative quantification in qPCR analysis as we previously reported and it is based on the critical cycle number (Ct) generated by qPCR analysis [37]. Statistical significance of the differences among groups in MTS proliferation assays and qPCR analysisdata was compared by two-tailed t test or one-way analysis of variance (ANOVA) using GraphPad Prism 5 (Version 5.04; GraphPad Software, La Jolla, CA, USA). Differences were considered statistically significant when the relevant *p* values were <0.05. 

## 5. Conclusions

This is the first study to show that the downregulation of *IGFBP5* in ESCC cells is closely associated with cisplatin-resistance. Knockdown of *IGFBP5* in parental SLMT-1 cells confirmed that *IGFBP5* suppression is one of the mechanisms for ESCC cells to acquire cisplatin-resistance. And the cisplatin-resistance phenotype can be reversed by upregulation of *IGFBP5*.

## Figures and Tables

**Table 1:** Differential genes expression in SLMT-1/CDDP1R and SLMT-1.

Probe Set ID	Gene Title	Fold-Change
Genes downregulated in SLMT-1/CDDP1R		
211959_at	*IGFBP5*, insulin-like growth factor binding protein 5	−43.48
212671_s_at	*HLA-DQA2*, major histocompatibility complex, class II, DQ alpha 2 (multiple annotations exist)	−22.45
201117_s_at	*PE*, carboxypeptidase E	−16.43
236297_at	*CDNA FLJ45742fis*, clone KIDNE2016327	−14.09
Gens upregulated in SLMT-1/CDDP1R		
1557636_a_at	*LINC00520*, long intergenic non-protein coding RNA 520	16.89
219836_at	*SLITRK6*, SLIT and NTRK-like family, member 6	13.75
205830_at	*LOC100506377*, uncharacterized LOC100506377	8.91
210229_s_at	*COL15A1*, collagen, type XV, alpha 1	8.79

## Abbreviation

ESCC, esophageal squamous cell carcinoma; IGFBP5, Insulin-like growth factor binding protein 5; CDDP, cisplatin; DMSO, Dimethylsulfoxide; PBS, Phosphate buffered saline; FBS, fetal bovine serum; MEMα, minimum essential medium α; MTS, 3-(4,5-dimethylthiazol-2-yl)-5-(3-carboxymethoxyphenyl)-2-(4-sulfophenyl)-2*H*-tetrazolium; RT-PCR, Reverse transcription polymerase chain reaction; qPCR, quantitative PCR; RNAi, RNA interference; siRNA, small interfering RNA.
